# Histological evidence for reversible cardiomyocyte changes and serum cardiac troponin T elevation after exercise in rats

**DOI:** 10.14814/phy2.13083

**Published:** 2016-12-21

**Authors:** Jinlei Nie, Keith George, Fei Duan, Tomas K. Tong, Ye Tian

**Affiliations:** ^1^ School of Physical Education and SportsMacao Polytechnic InstituteMacaoChina; ^2^ Research Institute for Sport and Exercise SciencesLiverpool John Moores UniversityLiverpoolUnited Kingdom; ^3^ College of Basic Medical SciencesHebei UniversityHebeiChina; ^4^ Dr. Stephen Hui Research Centre for Physical Recreation and WellnessDepartment of Physical EducationHong Kong Baptist UniversityHong KongChina; ^5^ China Institute of Sport ScienceBeijingChina

**Keywords:** Cardiac biomarker, exercise, myocardium

## Abstract

This study characterized cardiac troponin T (cTnT) appearance and associated histological evidence of reversible or irreversible changes in myocardial ultrastructure, determined via electron microscopy, in rats undertaking isoproterenol (ISO) infusion or an endurance exercise challenge. Male rats were randomized into ISO and exercise groups. In ISO trials rats were killed 5 h (ISO‐5H) and 24 h (ISO‐REC19H) after a single ISO or saline injection (SAL‐5H; SAL‐REC19H). In the exercise trials rats were killed before, as a control (EXE‐CON), immediately after (EXE‐END5H) and 19 h after (EXE‐REC19H) a 5‐h bout of swimming with 5% body weight attached to their tail. Serum cTnT was quantified by electrochemiluminescence, and myocardial samples in ISO‐REC19H, EXE‐REC19H and SAL‐REC19H were harvested for assessment of specific mitochondrial injury scores using electron‐microscopy. cTnT was undetectable in all control animals (SAL‐5H/SAL‐REC19H and EXE‐CON). cTnT increased in all animals after ISO and exercise but the response was significantly higher (*P *<* *0.05) at ISO‐5H (median [range]: 2.60 [1.76–6.18] *μ*g · L^−1^) than at EXE‐END5H (median [range]: 0.05 [0.02–0.14] *μ*g · L^−1^). cTnT returned to baseline at EXE‐REC19H, but had not completely recovered at ISO‐REC19H (median [range]: 0.17 [0.09–1.22] *μ*g · L^−1^). Mitochondrial “injury scores” were significantly higher (*P *<* *0.05) in ISO‐REC19H compared to EXE‐REC19H and SAL‐REC19H, with no difference between EXE‐REC19H and SAL‐REC19H. Mitochondria from EXE‐REC19H appeared aggregated in nonlinear clusters in a small number of scans. These findings suggest that acute exercise‐induced appearance of cTnT in this animal model is only associated with reversible changes in cardiomyocyte structure.

## Introduction

Cardiac troponins (cTn: cTnT and cTnI) are highly specific and sensitive markers of myocardial cell damage and have been adopted as the gold standard for the biochemical detection of cardiomyocyte insult during acute myocardial infarction (AMI) (Collinson et al. 2003). Somewhat counterintuitively numerous studies have demonstrated that prolonged exercise, such as marathon running, can result in cTn appearance during exercise or recovery (Middleton et al. 2008; Shave et al. 2010; Gresslien and Agewall 2016). Postexercise cTn levels have risen above the clinical threshold values used to identify AMI in a number of studies (Shave et al. 2010; Gresslien and Agewall 2016) presenting something or a diagnostic dilemma.

The clinical interpretation of postexercise cTn release is complex, but should be interpreted holistically with other clinical signs and symptoms of cardiovascular disease. Despite this, case reports of sudden cardiac death in athletes with adverse cardiac structural remodeling as well as reports of links between cTn elevation and depressed cardiac function (La Gerche 2013; Eijsvogels et al. 2016) have prompted ongoing debate about the cardiovascular consequences of high levels of exercise exposure (Whyte et al. 2009). Whether exercise‐induced cTn release reflects reversible or irreversible changes in cardiomyocyte integrity is not clear and is currently the source of some debate (George et al. 2011; Guasch and Nattel 2013; Eijsvogels et al. 2016).

Evidence to determine whether exercise‐induced cTn release is associated with reversible or irreversible cardiomyocyte damage is not available in humans. Histological changes in cardiomyocyte structure, associated with elevated serum cTn, can be determined in animal models (Chen et al. 2000) in association with the application of controlled exercise doses. To our knowledge, Chen et al. (2000) and Olah et al. (2015) are the only two groups to synchronously evaluate histological changes in cardiomyocyte structure and postexercise serum cTn levels in animal experiments. These studies reported an elevation in cTnT as well as hematoxylin–eosin staining evidence of sporadic cardiomyocyte damage after prolonged exercise. Despite this the authors admitted that the subtle nature of myocardial injury may not always be identifiable by limited light microscopic examination (Chen et al. 2000; Olah et al. 2015). The authors also concluded that more detailed studies are required as there is a clear diagnostic and prognostic distinction between irreversible and reversible myocardial injury to cardiomyocytes. Detailed electron microscopy has been commonly used to identify myocardial injury in animal experiments (Ishikawa et al. 1997) and isoprenaline (ISO) infusion is a common experimental model of irreversible cardiomyocyte injury (Goldspink et al. 2004). Similarly this model has been employed to explore the relationship between cTn release and histological changes in cardiomyocyte structure in rats (Acikel et al. 2003).

Consequently, the purpose of this study was to observe cTnT appearance and histological evidence of reversible or irreversible damage to the structure of cardiomycoytes in a rat model of prolonged exercise in comparison to a standard ISO infusion protocol. Data were also compared to control data (prior to exercise and with saline infusion) and data were assessed immediately and after 19 h of recovery postexercise. The study of cTnT but not cTnI ensures comparability between our results and those of other related studies, given only a single cTnT assay from diagnostic manufacturer (Rowland et al. 2010) is commercially available (Shave et al. 2007).

## Methods

### Animals

Sixty‐six male Sprague–Dawley (SD) rats (12 weeks old), weighing 210–230 g, were used in these experiments. The rats were housed in cages in rooms regulated for temperature (21–23°C), humidity (40–55%), and light cycle (0700–1700) and provided laboratory rat chow and water ad libitum. The research involving rodents in this study conforms to the National Institutes of Health *Guidelines for the Care and Use of Laboratory Animals* and was approved by the Institutional Animal Care and Use Committee.

### Experimental protocol

The rat ISO‐treated protocol and exercise model were performed according to Goldspink et al. (2004) and Chen et al. (2000), respectively. All sessions for ISO and exercise groups started at 1000 h. On an experimental day, food was removed at 0800 h and returned to provide the rat chow and water ad libitum from 1500 h. The ISO groups consisted of 36 rats, which were treated with a single dose of either normal saline (10 mL · kg^−1^ body weight, controls; *n* = 16), or ISO (5 mg · kg^−1^ body weight; *n* = 20) injected subcutaneously into rats. The rats in the ISO trial were killed after 5 h (ISO‐5H, *n* = 8) and 24 h (ISO‐REC19H, *n* = 8) after injection. Four rats were removed from this protocol due to early death. The control rats were killed 5 h (SAL‐5H, *n* = 8) and 24 h (SAL‐REC19H, *n* = 8) after injection. The exercise groups consisted of 30 rats: eight rats were used as nonexercise controls (EXE‐CON) and killed at rest, 22 rats swam for 5 h in a swimming tank (temperature 35°C, depth 30 cm) with 5% body weight attached to their tail. Rat swimming with a 5% load is considered a high intensity exercise (>80% *V*O_2max_) (McArdle and Montoye 1966; McArdle 1967). A 180 liters water tank was divided into four lanes with a surface area of 30 × 30 cm and a water depth of 35 cm per lane to allow individual swim exercise, and the water maintained at 35°C. The rats maintained continuous swimming involves continuous movement of the rats’ forelimbs and hindlimbs while maintaining its snouts above the waterline. Six rats failed to complete the swim protocol. After swimming, the rats were towel dried and killed immediately (EXE‐END5H, *n* = 8), and 19 h after swimming (EXE‐REC19H, *n* = 8).

It has been shown that peak serum cTnT occurs at 4–6 h after ISO injection in rats (O'Brien et al. 2006). Similarly, serum cTnT concentrations in animals (Chen et al. 2000) and humans (Nie et al. 2011a; Tian et al. 2012) peaked ~5–6 h after the initiation of exercise, independent of duration. Consequently we believe we employed a suitable design to obtain coincident peak comparisons of serum cTnT by drawing blood samples 5 h after ISO injection or the initiation of exercise. In addition, given that the peak histological severity (myodegeneration and necrosis) does not typically occur until 18–24 h after exercise (Chen et al. 2000) or ISO injection (Ishikawa et al. 1997; Goldspink et al. 2004), hearts from EXE‐REC19H (*n* = 8) and ISO‐REC19H (*n* = 8), along with controls (SAL‐REC19H, *n* = 8), were selected for histological examination.

### Tissue preparation

Rats were anesthetized with sodium pentobarbital (Nembutal, 50 mg · kg^−1^ ip). The abdominal cavity was quickly opened and ~5 mL of blood was drawn from the abdominal aorta. Serum samples were collected and stored at −80°C for cTnT measurements. The chest cavity was then quickly opened and the heart was excised. Left ventricular tissues were removed and immediately placed in glutaraldehyde.

### Biochemical assays

For analysis of serum cTnT, a third‐generation assay was performed using an electrochemiluminescence technology employed by the Elecsys 2010 automated batch analyzer (Roche Diagnostics, Basel, Switzerland). This assay has been previously validated for use in several laboratory animals, including rats (Fredericks et al. 2001). The interassay coefficient of variation was 10% at 0.03 *μ*g · L^−1^ and 3% at 0.10 *μ*g · L^−1^. The intraassay coefficient of variation was 8% at 0.03 *μ*g · L^−1^ and 2% at 0.10 *μ*g · L^−1^, with a detection limit of 0.01 *μ*g · L^−1^ (which coincides with the 99th percentile value) and an upper limit of 25 *μ*g · L^−1^. Serum cTnT reported as <0.01 *μ*g · L^−1^ was represented as 0.005 *μ*g · L^−1^ (Cleave et al. 2001; Nie et al. 2011b). All cTnT analyses were performed with the same assay kit. Before the assays were performed, the analyzers were calibrated with standard calibrators according to the manufacturer‐recommended protocols.

### Histological examination

Myocardial samples for electron microscopic examination were minced into blocks (~1 mm per side), fixed in glutaraldehyde (30 mL · L^−1^ in 0.1 mol · L^−1^ sodium cacodylate buffer, pH 7.4), postfixed in 10 g · L^−1^ osmium tetroxide, dehydrated in ethanol, embedded in Spurr's low‐viscosity resin, and sectioned with a Sorvall Porter‐Blum ultramicrotome (Leica Co., Wetzlar, Germany) and a diamond knife. Preliminary sections were stained with alkaline toluidine blue, and appropriate areas were chosen for sectioning. Random sections were collected on 200‐mesh copper grids, stained with uranyl acetate and lead citrate, coated with carbon, and examined in an electron microscope (JEM‐1230, JEOL Ltd, Tokyo, Japan).

According to the previous work (Ishikawa et al. 1997; Cheville 2009), mitochondrial quality is the key to distinguishing reversible and irreversible cell injury. Specifically, intact mitochondria are characteristic of reversible changes, whereas irreversible changes included disintegration of mitochondrial cristae and matrix proteins and accumulation of large dense granules accumulate in the matrix. These changes can lead to cell death if critical numbers of mitochondria are affected (Cheville 2009). A mitochondrial “injury scoring” system was adapted from Rahman et al. (1982) and Cheville (2009). Evaluation was made of 3+ blocks per heart and scoring was tabulated individually for each of 20 grid squares of tissue section using the criteria presented in Table 1. A severity and extent score was produced for each grid square for each rat. The pathologist scoring the sections was blind to the groups.

**Table 1:** Severity and extent scoring of mitochondria injury

Scores	Features
Severity scoring^a^	
0	Intact double membrane, compact orderly christae, and a homogeneous dense matrix
1	Mitochondrial swelling
2	Lysis and breakage of mitochondrial cristae (i.e., cristolyis)
3	Mitochondrial matrix proteins disintegrate
4	Large dense granules in the mitochondrial matrix
5	Ruptured and fragmented mitochondria
Extent scoring	
0	Fully intact
1	Involvement of single scattered myofibers
2	Involvement of groups of myofibers
3	Focal groups
4	Confluent groups

a The pathological descriptors of each ascending severity score are additional features to those of the lower scores.

### Statistical analyses

The Kolmogorov–Smirnov test was used to evaluate the normality of the data distribution. Due to the skewed distribution of cTnT data were expressed as median and range and we applied a nonparametric Kruskal–Wallis *H* test to compare the serum cTnT across time points. A Mann–Whitney *U*‐test was applied post hoc to assess pairwise comparisons where appropriate. The rates of cTnT‐positive (the percentage of subjects with cTnT exceeding the detection limit of 0.01 *μ*g · L^−1^) at each assessment point were compared using Fisher's exact test. One‐way analysis of variance (ANOVA) was employed to examine the differences in histological mitochondrial “injury scoring” among ISO, exercise and control groups. Post hoc analyses using Newman–Keuls were performed for cases in which the main effect was significant. Statistical significance was assumed at a level of *P *<* *0.05. Data analysis was performed using the statistical software package SPSS 11.5 (SPSS Inc., Chicago, IL).

## Results

**Figure 1:**
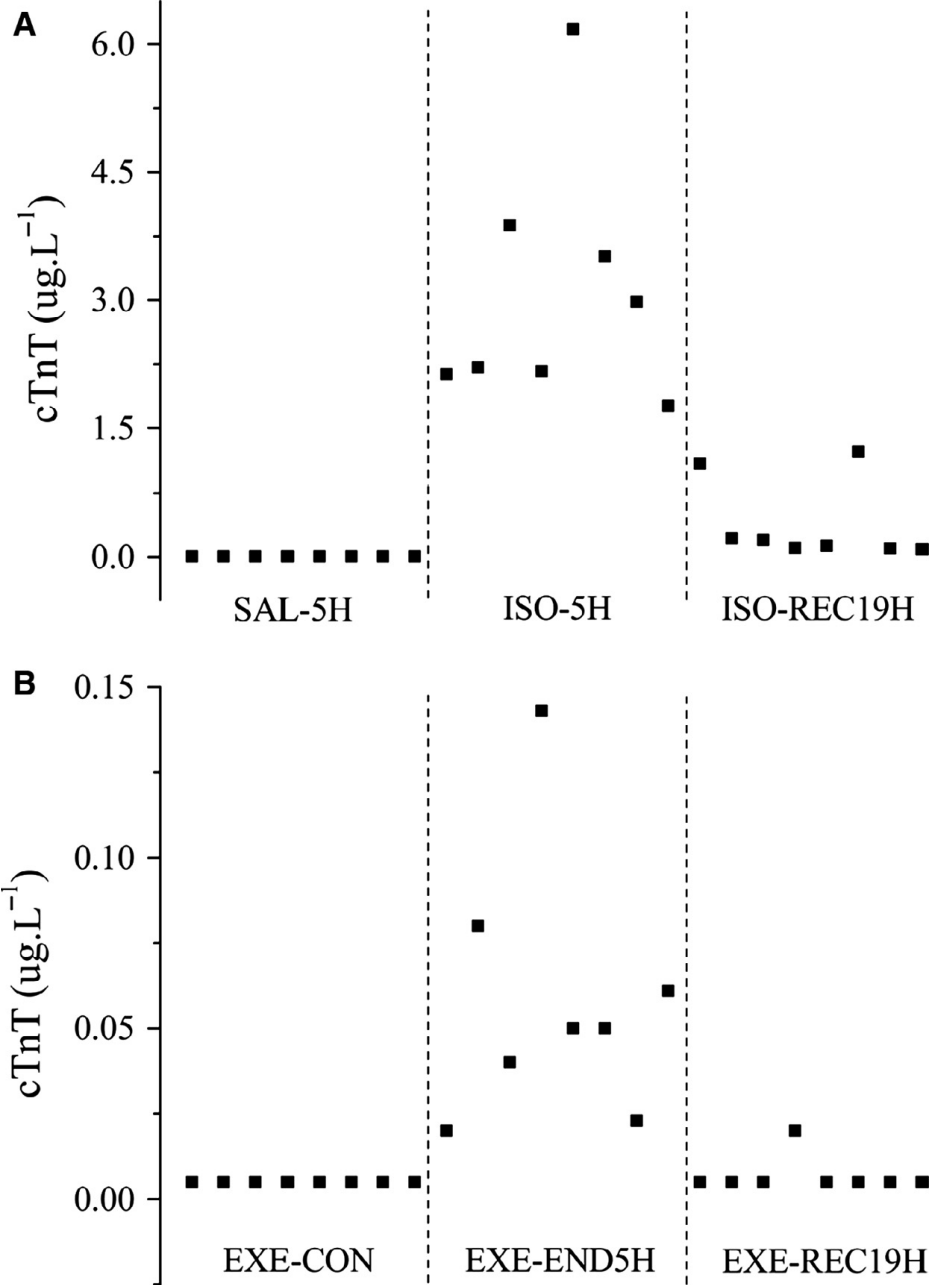
Individual data points for cardiac troponin T (cTnT) in isoprenaline‐treated (A) and exercise (B) rats.

Data for serum cTnT in ISO/Saline trials and exercise are presented in Table 2 and as individual data points in Figure 1. cTnT was undetectable in all animals at SAL‐5H/SAL‐REC19H and EXE‐CON. Serum cTnT was significantly (*P *<* *0.05) increased in all individuals, and as a cohort compared to respective controls, at ISO‐5H and EXE‐END5H, but the response was significantly higher (*P *<* *0.05) at ISO‐5H. Thereafter, cTnT returned to baseline at EXE‐REC19H (vs. EXE‐CON *P *>* *0.05), but not completely at ISO‐REC19H. Correspondingly, the positive rate of cTnT was significantly higher at ISO‐REC19H than EXE‐REC19H (*P *<* *0.05).

**Table 2:** Serum cardiac troponin T in ISO‐treated and EXE rats 5 h (5H) and 24 h (recovery) after the onset of intervention (ISO injecting or exercise), and corresponding CON

	CON	5H	Recovery
ISO			
Median (range, *μ*g · L^−1^)	0.005 (–)	2.60 (1.76–6.18)^a^ ^b^	0.17 (0.09–1.22)^a^ ^b^
Positive rate^c^	0/8	8/8^a^	8/8^a^ ^b^
EXE			
Median (range, *μ*g · L^−1^)	0.005 (–)	0.05 (0.02–0.14)^a^	0.005 (0.005–0.02)
Positive rate^c^	0/8	8/8^a^	1/8

ISO, isoprenaline; EXE, exercise; CON, control.

a Significantly different from corresponding CON value, *P *<* *0.05.

b Significantly different from corresponding EXE value, *P *<* *0.05.

c Values presented as number of positive/total observations.

**Figure 2:**
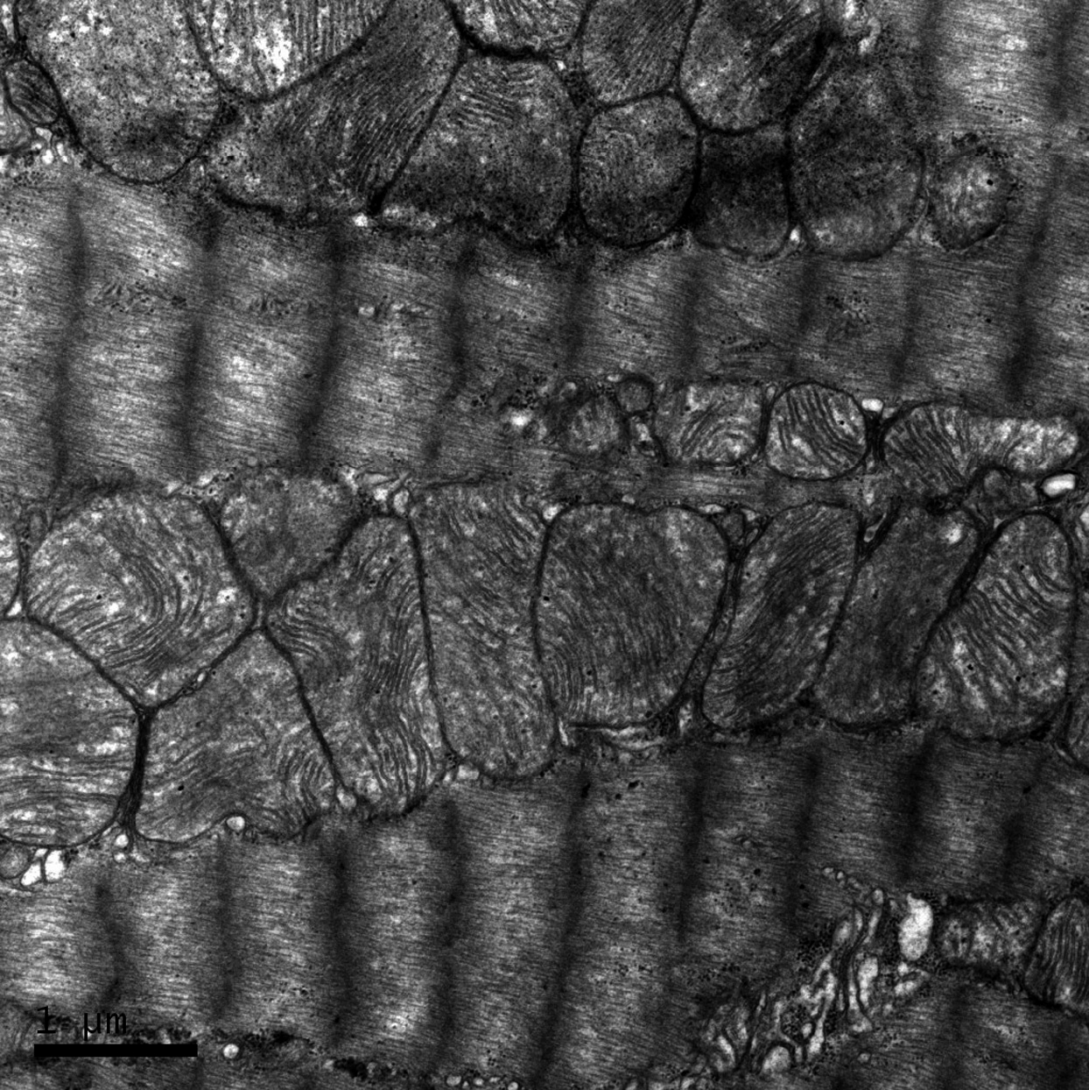
Electron micrograph of myocardium from a control rat showing intact mitochondria.

**Figure 3:**
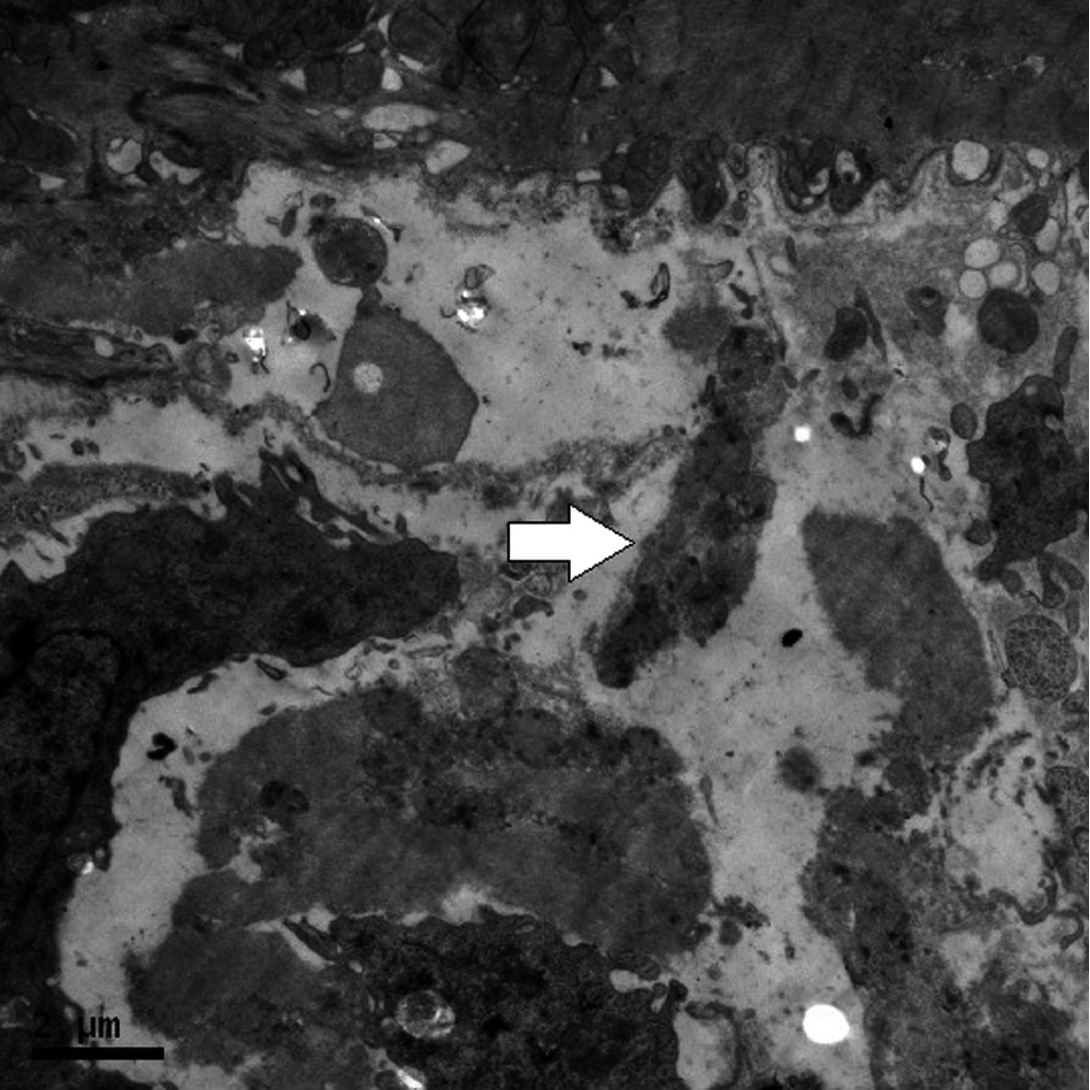
Electron micrograph of myocardium from an isoproterenol‐treated rat showing disrupted mitochondria (arrow).

**Figure 4:**
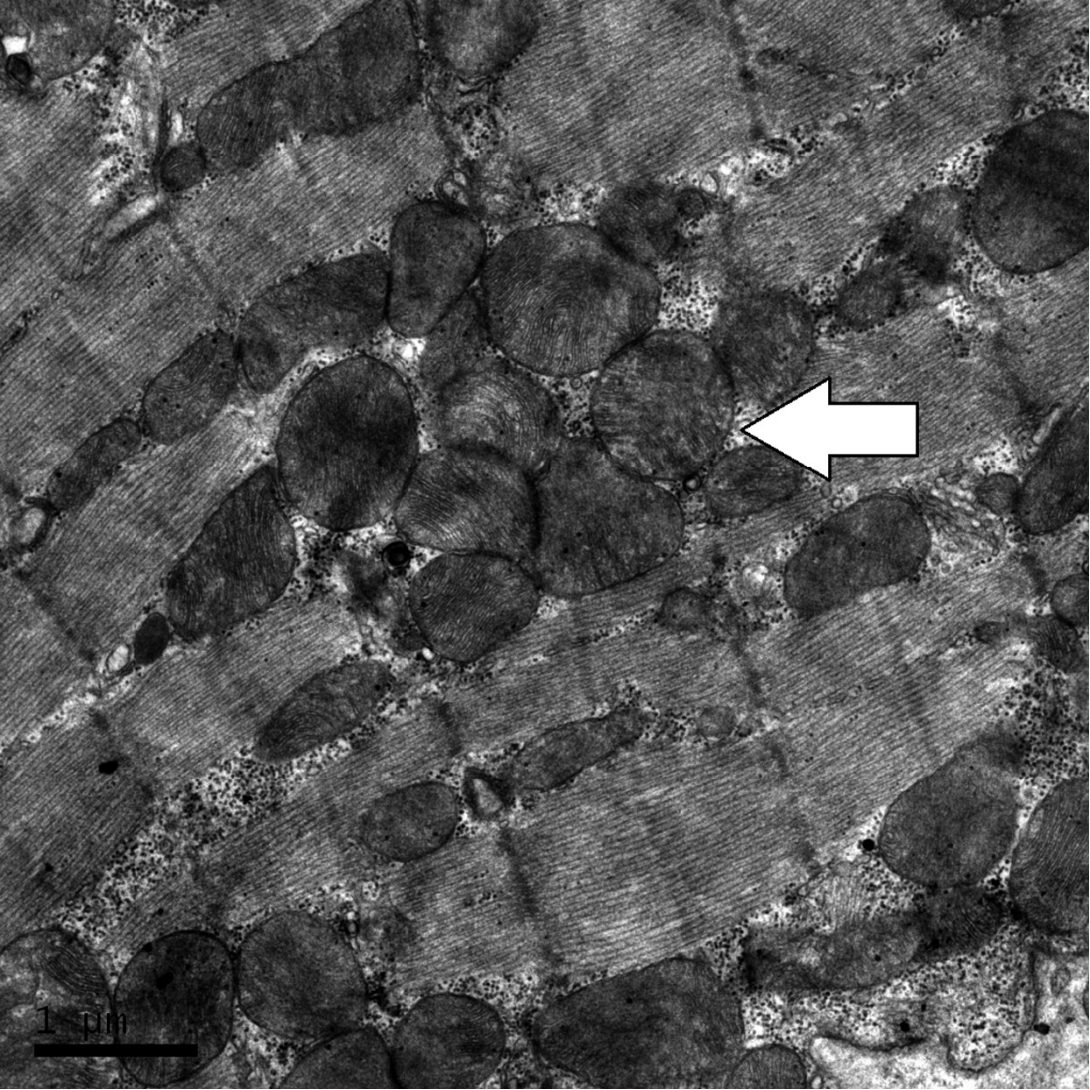
Electron micrograph of myocardium from an exercise rat showing intact mitochondia, but presence of mitochondrial aggregation (arrow).

Histological evidence in SAL‐REC19H showed mitochondria were intact (Fig. 2). Cardiac tissue from ISO treated rats (ISO‐REC19H) revealed disrupted mitochondria in both severity and extent when compared with the exercise (EXE‐REC19H) and control (SAL‐REC19H) groups (Table 3). The mitochondrial injury scores were not different between the EXE‐REC19H and SAL‐REC19H. The mitochondria from ISO‐REC19H samples demonstrated cristae and matrix proteins disintegration (scores 2–3), large dense granules accumulate in the matrix (score 4), and ruptured and fragmented mitochondrial remnants (score 5) (Fig. 3). The mitochondrial appearance of EXE‐REC19H was similar to that of SAL‐REC19H with only a small number of scans demonstrating mitochondrial aggregation in clusters (Fig. 4) and occasional mitochondrial swelling (score of 1).

**Table 3:** Electron microscopic evaluation of mitochondrial injury scoring (means ± SD, per gird)

Groups	Severity scores	Extent scores
ISO‐REC19H (*n* = 8)	3.31 ± 0.35	3.16 ± 0.33
EXE‐REC19H (*n* = 8)	0.18 ± 0.11^a^	0.18 ± 0.11^a^
SAL‐REC19H (*n* = 8)	0.11 ± 0.06^a^	0.11 ± 0.06^a^

ISO, isoprenaline; EXE, exercise; SAL, saline.

a Significantly different from corresponding ISO‐REC19H value, *P* < 0.05.

## Discussion

This study demonstrated that cTnT appeared in the circulation of rats after both a single bolus injection of ISO and 5 h of forced swimming. cTnT release was greater without full recovery at 24 h after ISO compared to exercise. Additionally, cTnT appearance after ISO was associated with histological evidence of irreversible cardiomyocyte damage. Uniquely, we observed that the cTnT appearance after 5 h of exercise in rats was not associated with histological evidence of irreversible cardiomyocyte injury.

### Effects of bolus ISO injection on serum cTnT and histological evidence of cardiomyocyte damage

Experimental injection of ISO in animals is a well‐established model to study the relationship between cardiac troponin release and histological changes associated with irreversible cardiomyocyte injury (Acikel et al. 2003). We confirmed both significant cTnT release and histological evidence of irreversible cardiomyocyte injury with ISO. The mitochondria from ISO‐REC19H samples presented with cristae and matrix proteins disintegration and/or accumulation of large dense granules in the matrix in focal groups of myofibers. Ruptured and fragmented mitochondria also appeared in some instances histological scans. Disrupted mitochondria shut down ATP production which leads to a state of irreversible injury (Cheville 2009). The mechanism(s) of ISO‐induced injury to myocardial cells might involve hypoxia, calcium overload and excessive production of free radicals (Acikel et al. 2003). In this model, the severity of the lesion in the myocardium is directly proportional to the dosage administered (Ferrans et al. 1969). Based on the effects of different dosages of ISO reported (Ferrans et al. 1969; Acikel et al. 2003; Goldspink et al. 2004; O'Brien et al. 2006), 5 mg · kg^−1^ body weight used in this study is the minimum dosage to produce irreversible cardiomyocyte injury in rats.

There is substantial evidence showing that blood levels of cTnT closely parallel the severity of myocardial injury (Collinson et al. 2003). This may explain the fact that, in this study, the median cTnT data following ISO injection were ~50 times above the response postexercise. The large difference in serum cTnT release probably reflects the release of cTnT from different “pools” within the cardiomyocytes. In ISO‐treated rats severe irreversible cardiomyocyte injury allows all cellular compartments containing cTn to contribute to elevated serum level. Furthermore, the delayed recovery of serum cTnT in ISO‐treated rats is consistent with a continuous liberation of cTnT from disintegrating myofibrils observed in other similar ISO‐treated animal studies (Goldspink et al. 2004).

### Exercise‐induced cTnT release and ultrastructural changes

The release of cTnT into the blood after prolonged exercise has been described earlier in our animal swimming models (Nie et al. 2010) as well as numerous human exercise studies (Shave et al. 2010; Eijsvogels et al. 2016). Middleton et al. (2008) observed cTnT elevations in all well‐trained men both during and after a treadmill marathon using multiple time point measurements. The fact that cTnT was present in the circulation of all rats at postexercise in this study supports the findings Middleton et al. (2008) and suggests an almost obligatory response/phenomenon.

The current data are the first to assess the association of exercise‐induced circulating cTnT elevation with histological changes in myocardium via electron microscopy. Our results demonstrated that no cardiomyocytes exhibited cristolyis and more severe mitochondrial injury in exercised rats. These findings are consistent with those of another early study, which used an exhaustive treadmill running model in rats (exercise duration: ~3 h) but reported no blood cTnT data (Maher et al. 1972). A small of fraction of mitochondria appeared swollen in exercise and control scans. Mitochondria are exquisitely sensitive to changes in homeostasis and the swelling that develops after stress may reflect the entry of water into the mitochondrial matrix (Cheville 2009). No differences in mitochondrial appearance were noted between the exercise and control group.

That fact that exercised rats displayed a lower cTn response compared to ISO rats seems to support the theory that only cytosolic cTnT (5% of total cTnT) “leak” were available to leak into the blood stream (Shave et al. 2010). The resolution of electron microscopy cannot identify small changes in membrane permeability and as such we cannot confirm the precise source of blood cTnT in exercise rats, and this requires further study.

It is important to note that the current conclusion is at odds with Chen et al. (2000) and Olah et al. (2015). Both studies noted that swimming exercise in rats resulted in histological evidence of myocarditis‐like changes in cardiac structure. Whether this simply reflects prolonged exercise‐induced immunosuppression with a presumed increased susceptibility for infection (Harris 2011) is not known. Based on the light microscopic data alone, however, it is difficult to draw any definitive conclusion on the nature of cardiomyocyte damage in these studies (reversible or irreversible) (Ishikawa et al. 1997; Chen et al. 2000).

### Implications

This study supports the notion that a postexercise cTn increase represents a physiological process that may include reversible changes in cardiomyocyte ultrastructure (George et al. 2011). The cTn elevations are modest and kinetic data suggest a rapid return to normal. In this regard, our data suggest that a cTnT response postexercise is common, if not obligatory, but is unlikely to reflect an irreversible insult to the cardiomycoytes in the absence of other elements of clinical disease or risk.

In addition, a reduction in cardiac function subsequent to prolonged exercise in healthy humans has been widely reported (Oxborough et al. 2010), which raise the question of whether acute prolonged exercise could produce persistent myocardial dysfunction (Shave et al. 2010). Our current findings do not support the possibility, at least on the cardiomyocytes basis. Nevertheless, care is still required when interpreting cTn data in patient assessment after significant exercise exposure. What is still not known is whether the highest cTnT values detected in human work (Nie et al. 2011b) represent a threshold for differentiating between reversible and irreversible myocardial changes in response to exercise.

Recently, Olah et al. (2016) showed differences in energy‐dependent early diastolic function and mechano‐energetics between long‐term exercise training‐ (physiological) and pressure overload‐induced (pathological) left ventricular hypertrophy could be explained by alterations in mitochondrial regulation, as reflected by normal mitochondrial biogenesis in physiological hypertrophy, whereas a marked mitochondrial dysfunction was observed in pathological hypertrophy. In this study, we support and extend the training work (Olah et al. 2016) by demonstrating the acute effects of intense exercise on mitochondria in heart. Specifically, the mitochondrial appearance of exercise rats was normal, and was similar to that of control rats. The results from this study, along with the Olah et al. (2016) work, seem to suggest that the differences between physiological (exercise) and pathological cardiac hypertrophy are doomed from the start. Moreover, recent evidence also showed that mechano‐energetics recovery contributes to myocardial reverse remodeling with reductions in load (Ruppert et al. 2016). Although myocardial adaptations to left ventricular assist devices (LVAD) support indicate substantial potential for structural, functional, and molecular plasticity within severely diseased hearts, sustained myocardial recovery after LVAD removal has been reported in only a small percent of cases, although higher rates may be possible (Hellawell and Margulies 2012). It was presumed that the loss of excessive myocardium to necrosis was important contributing factor; however, the validated evaluation index is lack (Hellawell and Margulies 2012). The acute differences in mitochondrial appearance observed in this study may serve as a starting point for future reverse remodeling studies.

### Limitations and future research

While our study provides novel insight into the nature of cardiomyocyte damage and cTnT release with exercise, several limitations should be considered and future studies are warranted. First, this is a single acute study with short recovery observation period and does not reflect the nature of exposure in a trained myocardium, such as in athletes completing lifelong endurance training, and will require prospective longitudinal studies for confirmation. Second, in this study, rats in the exercise groups were forced to swim for 5 h with 5% body weight attached to their tail. This model cannot be directly translated to humans although it does represent a significant physiological challenge to the animal. Our results cannot be directly extrapolated to the range of exercise undertaken by humans, which may involve repeated periods of demanding physiological stress with limited recovery, and probably in conjunction with extreme environmental conditions including hypoxia and/or high temperature. Third, the lack of parallel cardiovascular dynamics measurements limits further interpretation of the current findings, though the reduction in cardiac function subsequent to prolonged exercise has been described in numerous human exercise studies (Oxborough et al. 2010) as well as animal swimming models (Olah et al. 2015). Fourth, cTnI was not included in this study. Although our prior studies observed a high‐positive correlation between post‐exercise cTnT and cTnI values (Nie et al. 2008, 2011b), additional cTnI assessment is recommended for future research, given the potentially higher myocardial specificity of cTnI (Mair 1997). Finally, recently there is increasing evidence that intense endurance activity may particularly tax the right ventricle (RV), which seems more vulnerable to exercise‐induced damage or fatigue, as compared with the LV (La Gerche 2013). Therefore, additional RV assessment is recommended for future research.

## Conclusions

In summary, data from this study confirm that rats injected with a single bolus of ISO demonstrate substantial and sustained release of cTnT that is associated with severe and irreversible histological evidence of cardiomyocyte injury. Exposure of the animals to a substantial exercise stimulus resulted in cTnT appearance in all animals although the magnitude was blunted and recovery to baseline by 24 h had occurred. Importantly this study demonstrated that the elevation of cTnT postexercise was not associated with any electron microscopy based histological evidence of irreversible cardiomyocyte injury. We suggest that exercise‐induced changes in serum cTnT may be an obligatory physiological response but is not associated with irreversible cardiomyocyte damage.

## Conflict of Interest

None declared.
